# Bedside Extraction of a Rectal Foreign Body Using Hyperinflated Foley Catheter Traction: A Case Report

**DOI:** 10.7759/cureus.77136

**Published:** 2025-01-08

**Authors:** Matthias Barden, Derek Breiner

**Affiliations:** 1 Medical Education, University of California Riverside School of Medicine, Riverside, USA; 2 Emergency Medicine, Eisenhower Health, Rancho Mirage, USA; 3 Emergency Medicine, University of California Riverside School of Medicine, Riverside, USA

**Keywords:** clinical images, computed tomography abdomen, emergency medicine procedures, foley catheter, rectal foreign bodies, sexual activities

## Abstract

Bedside rectal foreign body (RFB) extraction can be a challenging task in the emergency department (ED) and may require operative or endoscopic management if unsuccessful. Many techniques have been described for RFB extraction including utilization of a Foley catheter. We present a case of a RFB successfully removed at the bedside using a hyperinflated Foley catheter after initial attempts using standard inflation volume were unsuccessful. This RFB case report highlights the bedside management of a difficult clinical situation and suggests a potential new modification to an established procedural technique.

## Introduction

Rectal foreign bodies (RFBs) have been estimated to be the cause of 1.9 emergency department (ED) visits per 100,000 people in the United States, with an increasing annual incidence [1]. The most common explanation for the presence of RFB is intentional insertion for sexual stimulation, although accidents, assaults, and ingestions are also reported. Men are much more likely than women to present with this complaint [2, 3].

Most cases of RFB can be managed at the bedside [3, 4]. Generally, laboratory analysis and imaging are obtained to exclude potential complications, such as colonic perforation or hemorrhage [5]. In the absence of these complications, bedside extraction may be attempted as long as the specific clinical situation or nature of the retained object does not pose a particularly high risk for complication [6]. Many techniques have been used to assist with bedside extraction of RFBs, largely depending on the shape and material of the object retained [4-17]. Many techniques involve repurposing medical devices such as ringed forceps, speculums, vaginal delivery vacuum devices, endoscopic snares, esophageal dilation balloons, and others [10-13]. Foley catheters are widely available and have been reported extensively in use for RFB extraction. By passing the Foley catheter beyond a RFB, it can be used both to provide traction and also to release any potential vacuum phenomenon that could be contributing to difficulty with removal. [2, 6, 14, 17].

In complicated cases, such as those with perforation, or hemorrhage, or in cases where bedside attempts have failed, specialist consultation will be required. Such cases can be managed in the operating room or gastrointestinal procedure suite with endoscopy or surgical removal.

## Case presentation

A 63-year-old male presented to our emergency department requesting assistance with the removal of a four-inch silicone ball that was retained within his rectum. He admitted to inserting the ball into his anus during a session of sexual self-stimulation. The object was a commercially available device designed for this activity. The ball had originally included an attached retrieval cord that he intended to use for the removal of the ball from the rectum. However, the cord had a mechanical failure, becoming detached from the ball during his attempts to remove the object. This prompted his visit to the emergency room.

He had a history of a similar experience, where he presented to our emergency department for retained RFB approximately one year prior. On that visit, a potato and an embedded metal bolt were retained in the rectum. He had added the bolt to the potato as a method to be able to self-extricate the potato but was unsuccessful due to the potato moving too far into the rectum for him to reach it for retrieval at home. On that occasion, he was taken to the operating room, where he underwent anoscopy under anesthesia. The potato was removed anally after being cut up into smaller pieces. 

The patient's medical history included deep venous thrombosis, atrial fibrillation, and hypertension. His social history included cocaine abuse. The patient was only taking apixaban. At the time of this visit, he reported that the silicone ball had been lodged inside the rectum for approximately 18 hours, but he was otherwise without complaint. He denied fevers or chills, rectal bleeding, or pain. Vital signs were within normal limits. The general physical exam was benign, including the abdomen being soft, non-tender, non-distended, and without rebound rigidity or guarding. On the rectal exam, a firm and smooth foreign body was palpable, consistent with the patient’s report. 

**Figure 1:**
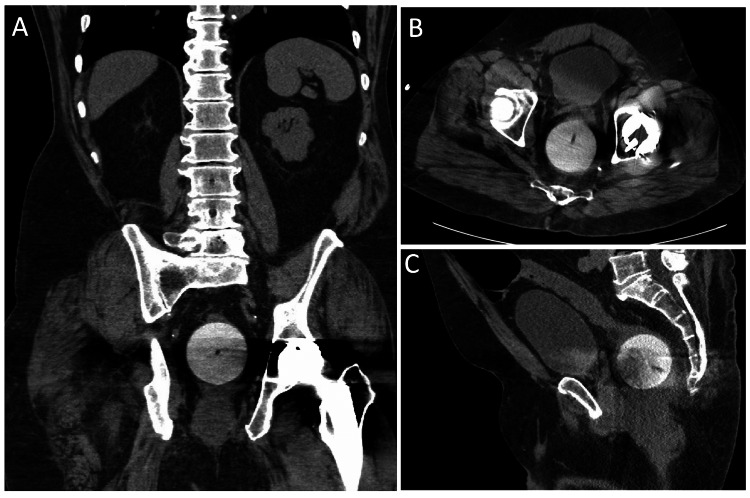
Representative computed tomography images of a silicon ball rectal foreign body seen in axial (A), sagittal (B), and coronal (C) projections.

He underwent a computed tomography (CT) scan of his abdomen and pelvis, as well as some basic laboratory analysis, including a complete blood count with differential analysis and a complete metabolic panel. Representative images from the CT scan are shown in Figure 1. The lab work was within normal limits. 

The patient agreed to attempts at bedside extraction of the silicone ball in the ED. The object could be easily palpated and even visualized just inside the anal verge but was too firm and slippery to easily grasp manually or with instruments. A 20 French latex three-way Foley catheter with a 30 mL balloon (Bardex Foley Catheter Ref 0167L20 CR Bard Inc Covington, Georgia) was lubricated and inserted into the anus and guided past the RFB. Initially, it was inflated to 30 mL with normal saline (NS) and used to pull traction on the RFB [18]. This was unsuccessful, as the Foley balloon tip slid past the RFB when significant traction force was attempted. 

**Figure 2:**
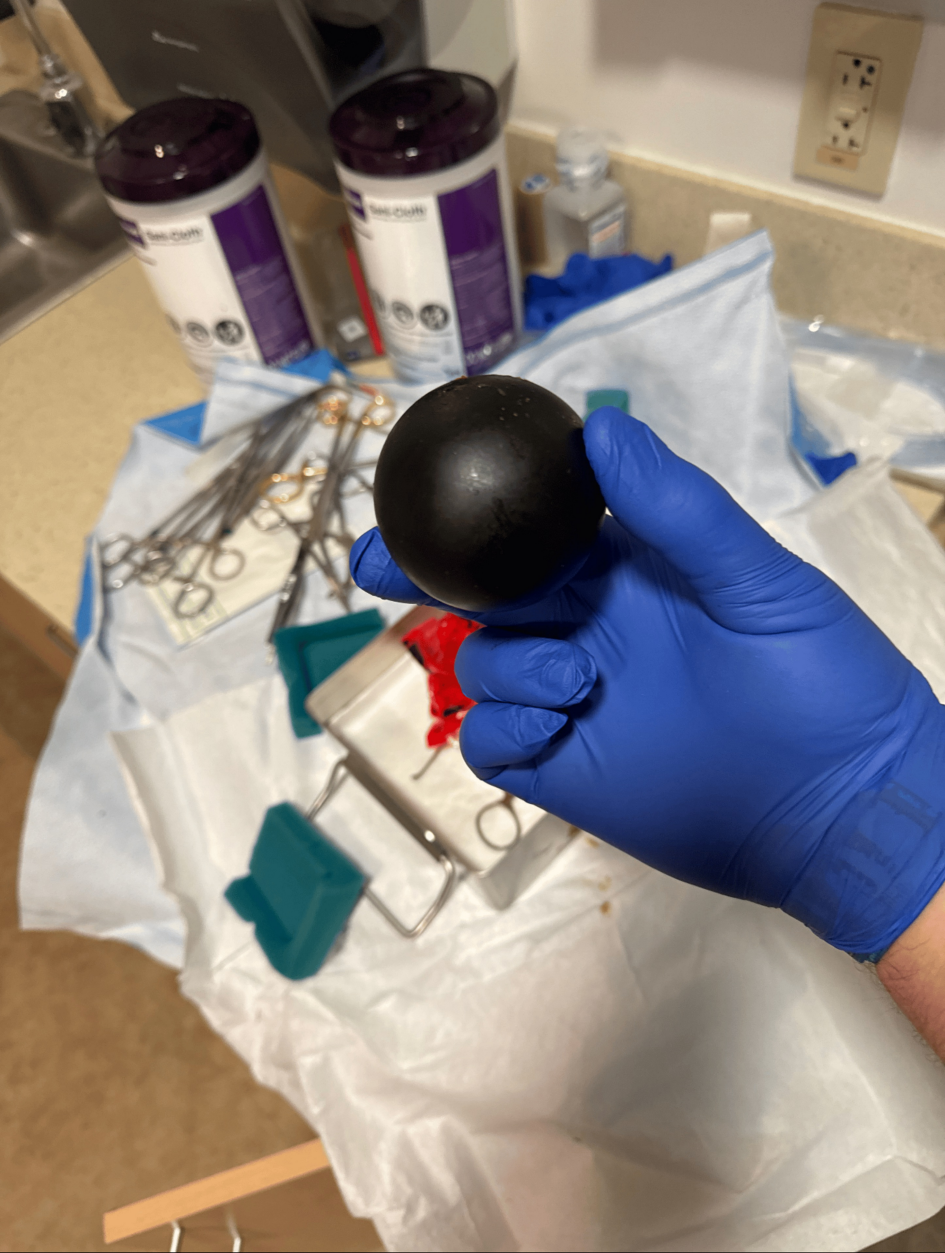
A four-inch-diameter silicone ball is pictured immediately after removal from the rectum using a hyperinflated Foley catheter for traction.

Based on prior cases of obstetrical and urological descriptions of Foley balloon hyperinflation for other clinical indications, the decision was made to attempt hyperinflation in this case. The Foley catheter was again inserted past the RFB, but this time was inflated to 60 mL with NS (double the standard recommended volume). With this larger volume balloon size, manual traction was sufficient to expel the RFB from the anus. No anesthesia or sedation was required in this case. A photo of the RFB after removal is presented in Figure 2. 

The patient was re-examined afterward and was without any evident skin tear or rectal bleeding. He was observed briefly and then discharged from the ED without any further complaints. He has not returned to our department since that time.

## Discussion

Bedside RFB extraction can be approached with many different techniques, largely depending on the size, shape, and material of the object [13]. Direct grasping was not possible in this case, given the firm, solid, and rounded nature of the object. Traction applied via a Foley catheter inflated to the recommended balloon volume of 30 mL was attempted but unsuccessful. Our impression was that the technique had promise, but that the 30 mL balloon size was insufficient to prevent the catheter from slipping past the object when traction was applied. We were aware of other clinical settings in which the Foley catheter balloon had been shown to accept much higher volumes than officially endorsed prior to bursting. By hyperinflating the same 30 mL Foley catheter to double the manufacturer-recommended volume, we were able to apply firmer traction and successfully remove the RFB at the bedside and avoid the need for operative management. 

The two most widely available Foley catheter balloon sizes are 5 mL and 30 mL. Larger sizes may be available for specialty applications but are generally avoided for normal urinary catheterization use due to complications that can result. These include bladder spasms and incomplete bladder drainage. Previous descriptions, found in the literature of RFB extraction using the Foley catheter technique, either did not specify the size of the catheter or used the 30 mL Foley catheter balloon size. There do not seem to be any prior reports of using Foley catheters inflated beyond 30 mL for this indication.

The ability of a standard Foley catheter to accept hyperinflation of the balloon has been described in the literature in various other clinical scenarios. Foley catheter volume has been studied in urological settings because hyperinflation to the point of rupture can be useful in cases of retained catheters with mechanical failures of deflation. In such studies, 30 mL latex catheters are noted to have burst volumes of over 400 mL [19, 20]. Foley catheters have also been repurposed for the obstetrical applications of inducing dilation of the cervix. In this application, hyperinflation to 80 mL was shown to be superior to 30 mL of balloon volume [21].

Based on the obstetrical and urological literature, hyperinflation of the Foley balloon was used in this case for RFB extraction. This added volume allowed for more forceful traction to be applied without causing the Foley balloon to slip around the side of the RFB. 

## Conclusions

Extraction of a retained RFB can be difficult or even impossible at the bedside. Many different techniques have been described to accomplish this procedure. The technique of choice depends on the type of object that is retained. While the Foley catheter technique may not function optimally for all types of RFB, it can be the best choice for objects that do not allow for traction to be applied otherwise. Foley catheters are widely available and have been extensively previously reported for use in RFB extraction. In cases where the standard Foley catheter inflation volume provides insufficient traction due to slipping past the retained object, hyperinflation is an additional possible augmentation of this technique. Successful removal a RFB at the bedside can potentially prevent patients from requiring operative intervention, avoiding its associated complications.
